# Retromode Imaging in Age-Related Macular Degeneration

**DOI:** 10.3390/medicina59040647

**Published:** 2023-03-24

**Authors:** Antonia-Elena Ranetti, Horia Tudor Stanca, Bogdana Tăbăcaru, Adrian Teodoru, Mihnea Munteanu, Simona Stanca

**Affiliations:** 1 Doctoral School, University of Medicine and Pharmacy “Carol Davila”, 020021 București, Romania; 2 Clinical Department of Ophthalmology, “Carol Davila” University of Medicine and Pharmacy, 020021 Bucharest, Romania; 3 Clinical Surgical Department, Faculty of Medicine, “Lucian Blaga” University Sibiu, 550169 Sibiu, Romania; 4 Department of Ophthalmology, “Victor Babes” University of Medicine and Pharmacy, 300041 Timisoara, Romania; 5 Clinical Department of Pediatrics, University of Medicine and Pharmacy “Carol Davila”, 020021 București, Romania

**Keywords:** age-related macular degeneration, retromode imaging, pseudo-three-dimensional, scanning laser ophthalmoscope, multimodal imaging

## Abstract

*Background and Objectives*: Retromode is a relatively new retinal-imaging technique that is based on the transillumination principle and is obtained with a scanning laser ophthalmoscope that uses light in the infrared spectrum. The laser light penetrates into the deep retinal layers and the choroid. Retromode images are captured with a laterally displaced aperture, and the detector captures only the scattered light. The result is a high-contrast pseudo-three-dimensional image. Age-related macular degeneration (AMD) is a disabling retinal disease. AMD is characterized in its early stage by small and intermediate drusen formation, while the signs of intermediate AMD are large drusen and/or pigmentary abnormalities. Late AMD has two forms, geographic atrophy, which is the advanced form of dry AMD, and wet AMD. Most of the lesions of AMD are located in the outer layers of the retina. This new imaging method can provide a glimpse of the deep retinal layers’ topographic changes in a non-invasive, fast, and effective way that can match the other imaging tools available. *Materials and Methods*: The literature review was performed by searching the PubMed database using the following combination of keywords: retromode imaging and age-related macular degeneration. Relevant images similar to the ones in the literature were identified and used as models. *Results*: The purpose of this article is to highlight the utility of incorporating retromode imaging into the multimodal evaluation of the retina in patients with AMD and to gather and integrate these findings into a brief but comprehensive paper. *Conclusions:* Retromode imaging is a good screening, diagnosis, and monitoring tool for patients with AMD.

## 1. Introduction

Age-related macular degeneration (AMD) is a retinal disease that causes central vision loss [1]. It is a public health issue because of the large number of affected patients, with 1.8 million cases in 2020. Following glaucoma, it is the second leading cause of irreversible blindness in adults over 50 years old [2]. AMD is a multifactorial disorder. The risk factors are age, female sex, white race, smoking, and low antioxidant intake [1]. Genetic factors involving the complement pathway and the age-related maculopathy susceptibility 2 locus (ARMS2) genes are usually present in most of the patients with late-stage disease [3].

Ferris et al. stated that normal aging changes in the retina consist of drupelets (small drusen), and these do not present an increased risk of developing the advanced retinal disease. According to the Beckman classification, early AMD is represented by medium drusen (>63 microns and <125 microns), while large drusen (>125 microns) and pigmentary abnormalities (hypo- or hyperpigmentation) are considered intermediate AMD. Late-stage disease is represented by its two variants of evolution: exudative (neovascular or wet) AMD or geographic atrophy (GA) [4].

The diagnosis of AMD was classically made based on clinical examination findings and relied on color fundus photography (CFP). Fluorescein angiography (FA) used to be the main investigation for treatment decisions [5]. Now the gold standard tool used by retinal specialists in the diagnosis and monitoring of the disease is spectral domain optical coherence tomography (SD-OCT), a non-invasive investigation that offers high-resolution cross-sectional images of the retina [6], which are used for detecting various biomarkers and classifications of AMD [7]. FA is still useful because it can confirm neovascularization, and it can also detect leakage, while newer methods such as optical coherence tomography angiography can not [8].

In the era of multimodality, fundus autofluorescence (FAF) is another non-invasive tool that proved to be very useful in detecting early and late AMD changes by assessing the lipofuscin quantity in the retinal pigmentary epithelium (RPE) [9,10]. Since technology is developing at a fast rate and there are newer methods to investigate AMD patients, it is worth turning a spotlight on retromode, another non-invasive imaging method of the retina.

## 2. Retromode Imaging

**Figure 1:**
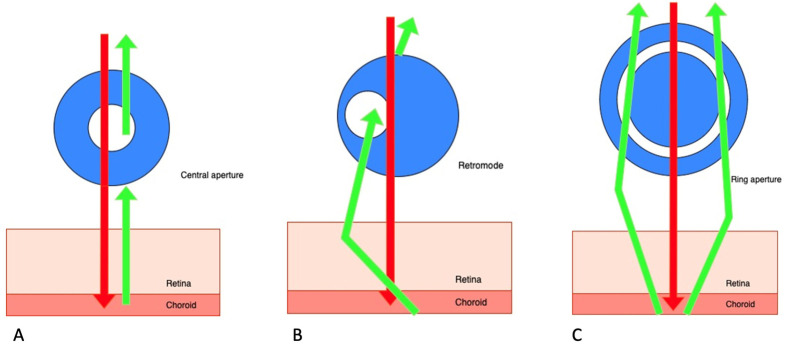
Scanning laser ophthalmoscope: three different aperture types: (**A**) confocal (with a central aperture). (**B**) retromode (with a laterally deviated aperture to the left or to the right), and, respectively, (**C**) the ring aperture.

Retromode is a relatively new imaging modality that uses the principle of transillumination [11]. The retromode images are captured with a scanning laser ophthalmoscope (SLO), which uses a laser light with a deviated aperture instead of a central aperture (see Figure 1) [11]. The aperture can be deviated laterally to the left (DL), to the right (DR), or annular (RA—using a ring aperture). The scattered light comes from the choroid and deep retina, and it does not return directly to the detector, so the result is a pseudo-three-dimensional (pseudo3D) image [11].

Images captured with a confocal SLO through a central aperture have good contrast and resolution. Both the reduced-size aperture, which is central in relation to the detector, and the scanning laser beam are focused on the same tissue location [11].

The latest system that can produce this kind of image is the Mirante SLO/OCT (Nidek Co., Gamagori, Japan). Retromode imaging was also available on an earlier version of this platform, the F-10 SLO (Nidek Co., Gamagori, Japan) system. The Mirante platform uses infrared laser illumination (790 nm) in order to capture the retromode images. The infrared laser light has the ability to penetrate deeper into the tissues than visible light and gets to the level of the outer retina layers, the RPE, and the choroid [11,12,13,14,15]. The lesions of the deep retinal and choroid tissues are highlighted with the shades of the laterally displaced light. The result is a high-contrast image obtained with DR and DL modes, which have the advantage of identifying more subtle abnormalities in the outer retina, the RPE, and the choroid. The images produced with the annular ring aperture are not as clear, and the contrast between structures is reduced [11].

The pseudo3D effect is in fact a virtual illusion that is induced by the shades and borders on one side of the structures illustrated in the images, which gives the observer a sense of the depth of the lesions [11,15].

### Retromode Images

The multimodal images of patients were taken in the clinic as a part of the standard testing protocol. The images were acquired with the Mirante SLO, which provides an image with a field of 40 degrees. The axial resolution of the confocal aperture is about 300 microns. Four separate laser wavelengths—infrared (790 nm), red (670 nm), blue (488 nm), and green (532 nm), coupled with a unique specialized sensor for each wavelength, were used to generate the color SLO images (cSLO) [11,16]. The SD-OCT images were also acquired with the Mirante platform. All the images were reviewed by one highly experienced retinal specialist in accordance with the diagnostic criteria, in order to identify similar and representative cases consistent with the ones already existent in the literature.

## 3. Age-Related Macular Degeneration Imaged with Retromode

Age-related macular degeneration is represented by heterogenous and pathognomonic manifestations, depending on the stage of the disease of the patient. Each of the clinical entities found in AMD will be described, along with the utility of this method in the diagnosis and monitoring of the patients found in literature, and illustrative images for the different clinical entities.

### 3.1. Early and Intermediate AMD

**Figure 2:**
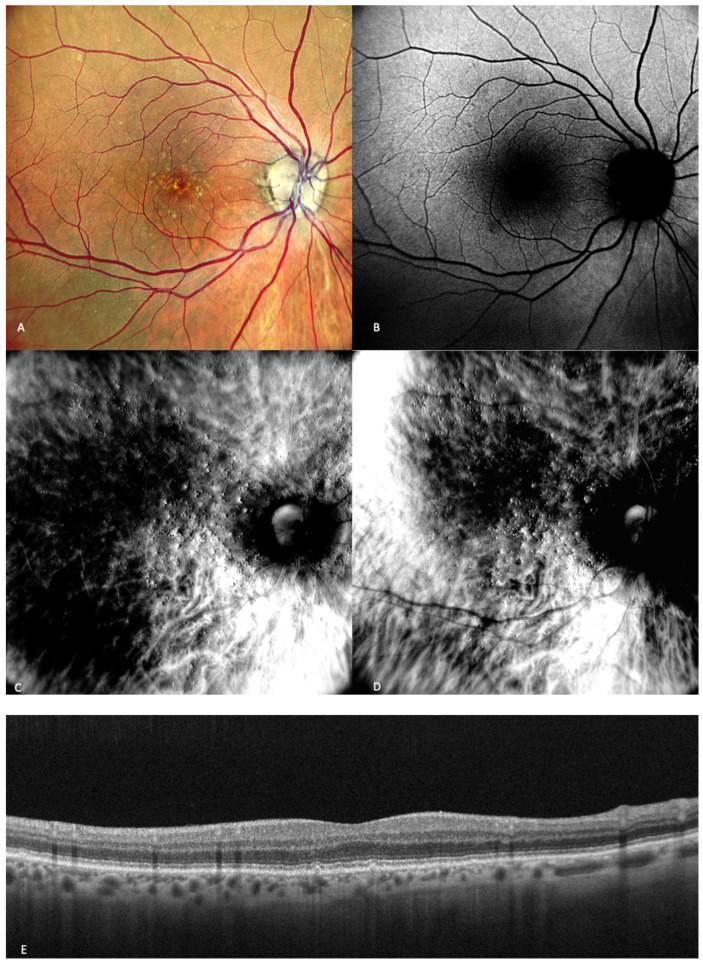
Early AMD. Multimodal imaging of small and medium drusen. (**A**) Color scanning laser ophthalmoscope photography shows multiple small drusenoid round, yellow deposits. (**B**) On the fundus autofluorescence, drusen hardly cause any effect. (**C**) Retromode deviated to right and (**D**) retromode deviated to left display drusen as small elevations or depressions, respectively. Drusen number and extension are obviously more easily visualized on the retromode images than on the cSLO image. (**E**) SD-OCT section, which shows drusen as small elevations of the RPE.

**Figure 3:**
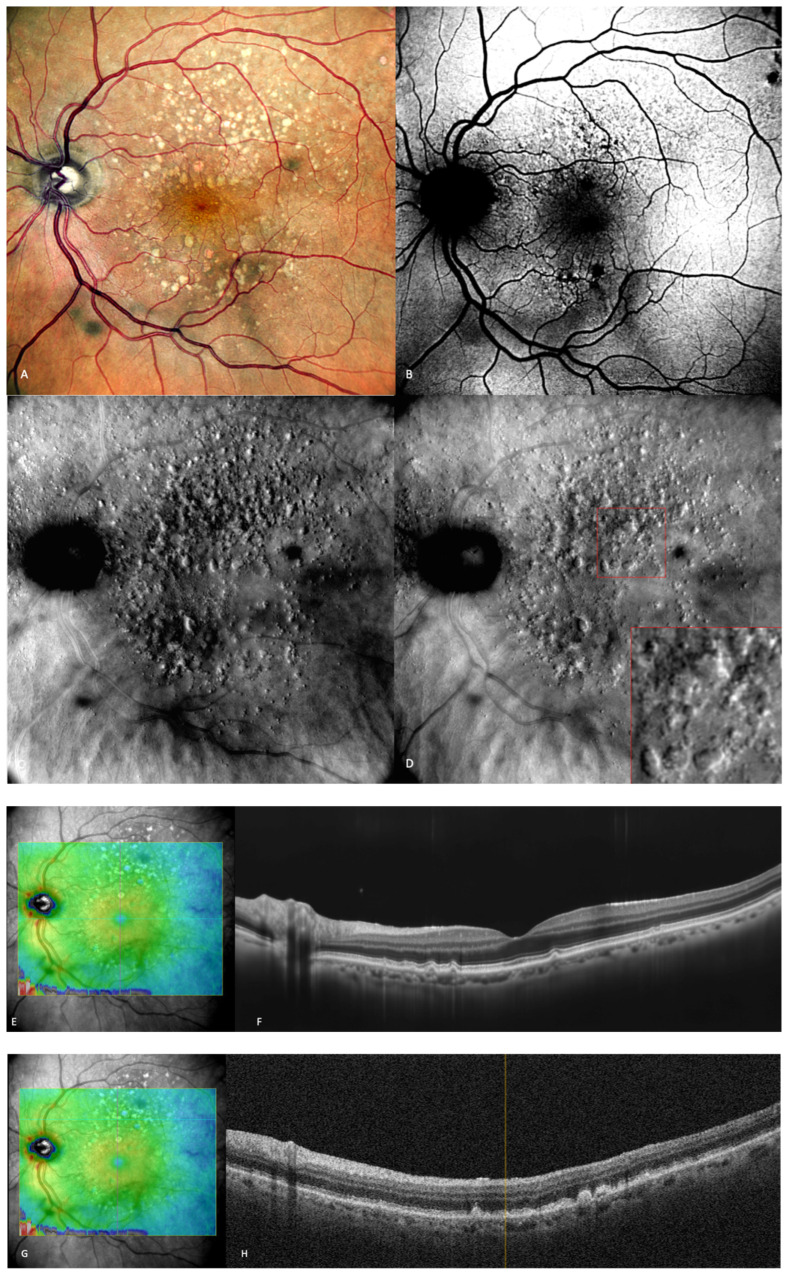
Intermediate AMD. (**A**) Multiple medium and large drusen can be seen on the cSLO image. (**B**) On green FAF, large drusen appear as hyper-autofluorescent patches. (**C**) Retromode deviated right depicting elevated, large drusen. (**D**) Retromode deviated left shows a “moon surface” appearance (zoomed image in the red square). (**E**,**G**) En-face OCT showing the location of the section in (**F**,**H**) SD-OCT showing multiple small, medium, and large drusen.

Early and intermediate AMD patients usually do not present any symptoms. Some of them may report mild image distortion when performing nearby tasks such as reading [1]. In early AMD (Figure 2), the main finding is medium-sized drusen. In patients with intermediate AMD (Figure 3), medium and large drusen appear, and also pigmentary abnormalities can be detected.

#### 3.1.1. Drusen

Drusen are incipient changes in the fundus that are indicative of age-related macular degeneration, and early identification is anticipated to become more crucial given potential therapies in the future.

Clinically, drusen show up as yellow-white deposits under the retina during biomicroscopy. The gold standard for determining the clinical stage of non-neovascular AMD has been color fundus photography (CFP) for a long time [17,18,19].

Drusen are identified by the buildup of extracellular material between the basal lamina of the RPE and the inner collagenous layer of Bruch’s membrane. Drusen can be classified depending on their consistency, size, and margin into four categories: hard drusen, soft drusen, cuticular drusen, and mineralized (calcified) drusen. Subretinal drusenoid deposits (also known as reticular pseudodrusen) are another entity, and they are localised above the RPE [18]. Large drusen are well-known to be a risk factor for developing advanced AMD [20,21,22], while hard drusen are not incriminated to be one.

Acton et al. conducted one study that assessed the utility of retromode imaging in the quantification of drusenoid deposits. They concluded that OCT scans did not show any tiny deposits smaller than 40 microns, which were attributed to the existence of minor lenticular opacities or unstable fixation. On OCT scans, larger drusen that were seen in fundus photography and retromode images showed up as a displacement of the RPE. The presence of drusen on OCT imaging was consistent with the size of the subretinal deposits seen in retromode imaging. The number of the detected drusen was significantly higher with retromode than with the drusen number on color fundus photography [13].

In one study, Diniz et al. sought to prove the efficiency of drusen detection by different SLO strategies and concluded that the hard drusen number detected with retromode illumination was almost twice the number detected with CFP [14]. In another study, Diniz et al. observed that for counting drusen number, the SD-OCT-derived RPE elevation map is inferior to other imaging modalities, such as CFP, and particularly to retromode. They stated that drusen appeared as raised lesions with clear boundaries, so it was easier to detect and count them more precisely on the retromode images, even though the mean drusen volume was not different [23].

A more recent study published in 2020 by Cozzi et al. aimed to determine the accuracy of different retinal-imaging modalities in detecting various retinal lesions in patients with early AMD. The study concluded that hard (small) drusen are very well-identified with retromode DR (92% sensitivity; 58.3% specificity) or DL (85.2% sensitivity; 83.3% specificity) [24]. Soft drusen were also detected by retromode with good accuracy: DR (78.5% sensitivity; 85.7% specificity) and DL (79.7% sensitivity; 81% specificity) [24].

Drusen are elevated structures, which were described to have a “moon surface” appearance (Figure 3) on the retromode pseudo3D images, whether pseudo-elevated (convex) when the dark shadows are deviated to the right side or pseudo-inverted (concave) when the light comes from the left side. The drusenoid deposits look like small and large mounds and/or depressions [13,14,25].

#### 3.1.2. Subretinal Drusenoid Deposits

**Figure 4:**
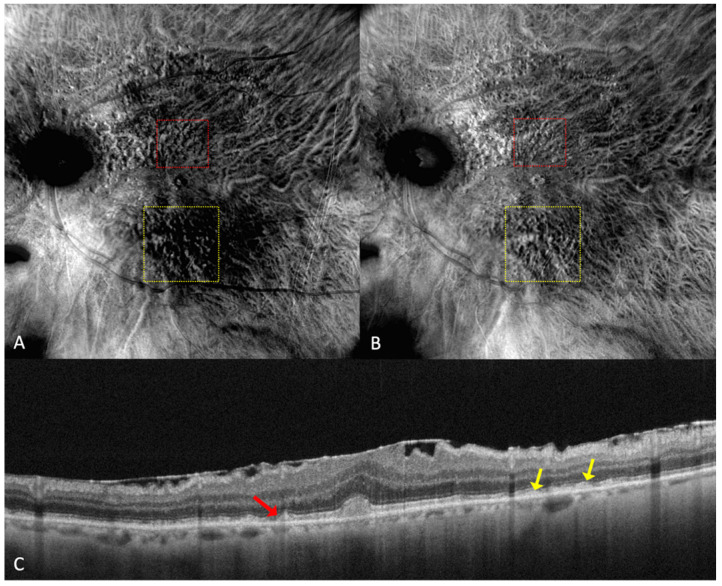
(**A**) On the retromode image DR dot SDDs are round, hyporeflective lesions, and ribbon SDDs are difficult to see. (**B**) Retromode image DL shows each dot SDD as “hyporeflective core” with a “hyperreflective halo” [24], while the ribbon SDDs tend to have a reticular structure. (**C**) SD-OCT shows dot SDD (red arrow), ribbon SDDs (yellow arrows), and also a hyper-reflective layer on the retinal surface.

**Figure 5:**
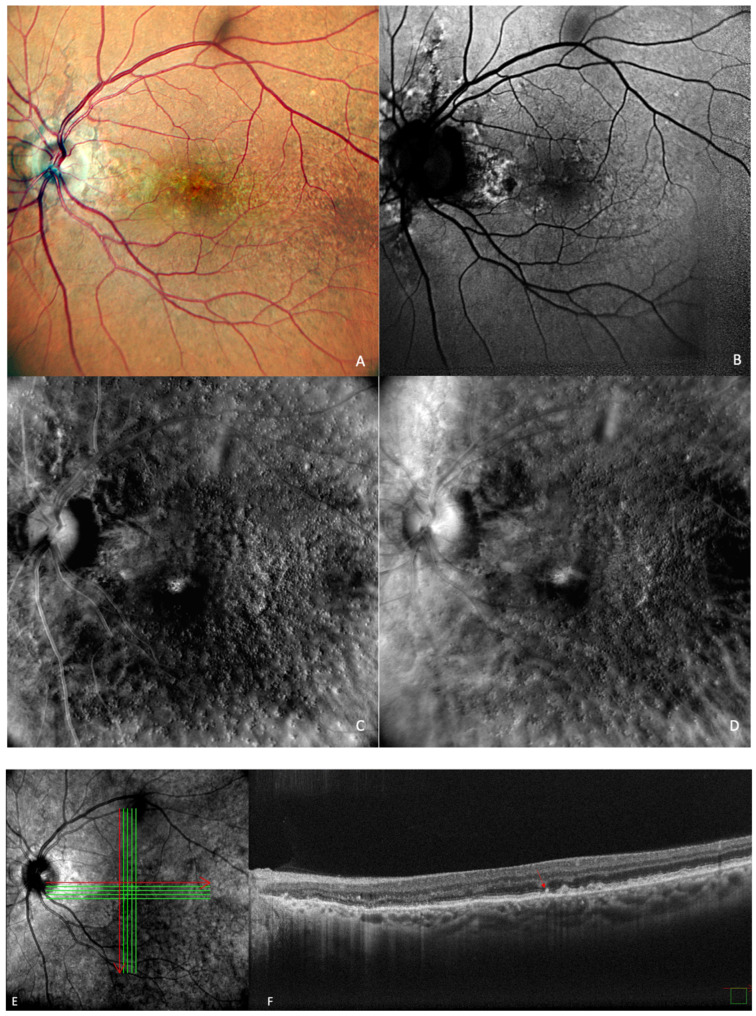
Drusen and dot subretinal drusenoid deposits (SDD) in a patient with co-existent angioid streaks: (**A**) cSLO showing multiple drusen and SDDs, with peripapillary radiating irregular lines. (**B**) In green FAF, temporal to the fovea, drusen are seen as hypo- and hyper-autofluorescent round lesions. (**C**) In retromode deviated right, elevated lesions can be seen, and in (**D**) retromode deviated left, small, depressed lesions are detected. (**E**) En face OCT of (**F**) SD-OCT shows multiple small, medium, large drusen and also dot SDD (red arrow).

Reticular pseudodrusen or subretinal drusenoid deposits (SDDs—see Figure 4 and Figure 5) are located above the RPE and are considered to be a high-risk factor for the progression of AMD to the late forms: GA or neovascularization [26,27]. SDDs have an inclination to appear superior to the fovea and spare the more central macula [28]. SDDs were classified by Suzuki et al. in three main subtypes: dot, ribbon, and midperipheral [29]. These entities are best detected with SD-OCT and with infrared reflectance, and it is recommended to use multiple imaging modalities in order to diagnose SDDs [30].

Parravano et al. conducted a study on reticular pseudodrusen imaging. Considering the characteristics of the lesions by their aspect on retromode images, the deposits were classified as round, bended, or interlacing [31].

Cozzi et al. investigated the accuracy of different retinal-imaging modalities in AMD patients. The study found that dot SDDs are hard to detect with retromode because of their high resemblance to small drusen, so the method’s accuracy is reduced in both DR (64.6% sensitivity; 85.7% specificity) and DL (76.6% sensitivity; 83.3% specificity), in comparison to other investigations [24]. The authors also specified that the DR or DL modes might not be able to detect ribbon SDDs due to their small size [24].

Dot SDDs in the retromode DR mode appear as pseudo3D round, hyporeflective lesions, and in the DL mode, they appear to have a target aspect—a hyperreflective halo surrounding a hyporeflective center. Ribbon SDDs are hardly detectable in the retromode illumination DR, and in the DL mode, they have a “reticular pattern” [24].

#### 3.1.3. Retinal Pigment Epithelium Abnormalities

Another significant characteristic of non-neovascular AMD is represented by RPE abnormalities, which indicate a higher likelihood of development into atrophy and NV [20,21,22].

Bruch’s membrane thickens and loses permeability as a result of drusen deposition in tandem with other structural and biochemical alterations related to AMD pathogenesis, such as chronic activation of the complement cascade and inflammation [32]. This obstructs both nutrient transfer to the retina and waste exchange to the choroid and is accompanied by thinning of the choroidal vasculature. In early or intermediate illness, these actions, along with neurodegenerative changes in the photoreceptor-RPE complex, cause pigmentary abnormalities of the RPE, including hypo- or hyperpigmentation [33]. In the normal aging eye, RPE cell counts decline, though this loss is much less than that experienced by individuals with AMD [34]. The RPE’s pigmentation and shape change as a result of an increase in lipofuscin granules and a decline in melanosomes [34]. These changes in the RPE pigment granules have been recently demonstrated in vivo with a new investigation method by Meleppat et al. [35].

Shin and Lee aimed to prove the utility of retromode in the detection of RPE alterations that appear in central serous chorioretinopathy (CSCR) and to describe the pathology captured using this method [36]. Retromode identified fine RPE alterations and subretinal fluid undetectable with other imaging techniques, such as FA [36].

Giansanti et al. compared the capacity of FAF and retromode to detect different lesions at the RPE level in CSCR [37]. The outer retinal lesion detections assessed in this study are: the presence of subretinal fluid (SRF), pigment epithelial detachment (PED), hyper-, iso- and hypo-autofluorecent RPE degeneration, and RPE atrophy. Most of the features listed above are also common in AMD. Retromode distinguished subretinal fluid (93%), pigment epithelial detachment (79%), RPE degeneration with iso-autofluorescence (100%), and hypo-autofluorescence (90%) very well. Retromode also detected RPE degeneration with hyper-autofluorescence (68%), and in more than half, it also identified RPE atrophy (57%). Retromode outperformed FAF at detecting SRF, PED, and also iso-autofluorescent degenerative lesions. RPE mottling and subtle, small RPE changes were very well-captured with retromode, while FAF could not detect these lesions [37].

The anatomical separation of the RPE from the underlying Bruch layer is what is known as RPE detachment [38]. PEDs in AMD can be categorized as either drusenoid, serous, vascularized, or mixed. Drusenoid PEDs are mostly characteristic of the dry or non-neovascular type. The neovascular or wet form of AMD is frequently associated with serous PEDs, but the natural history is generally more favorable. On the other hand, there is a considerable risk of vision loss in vascularized PEDs connected to Type 1 neovascularization and wet AMD [38].

In retromode, subretinal fluid is seen as a sharp, convex, round, three-dimensional, translucent protuberance. PED is detected as a clear, convex, circular or irregular, translucent, elevated lesion [36].

### 3.2. Late AMD

Patients with late AMD present with very reduced central visual acuity. End-stage AMD can be divided into two categories based on whether choroidal neovascularization is present or absent: non-neovascular (known as non-exudative, atrophic, or dry) or neovascular AMD (also referred to as exudative or wet) [1].

#### 3.2.1. Geographic Atrophy

**Figure 6:**
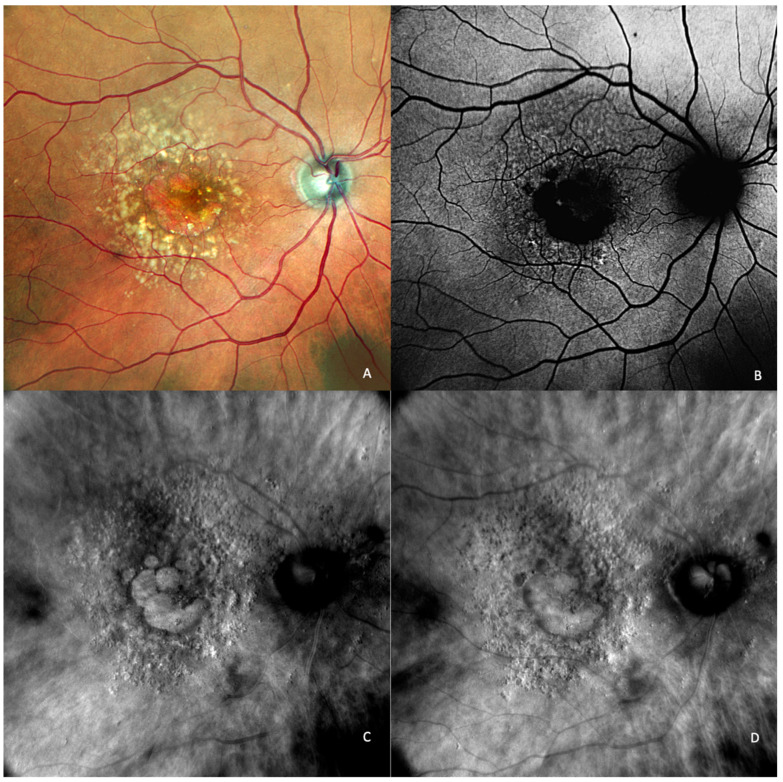

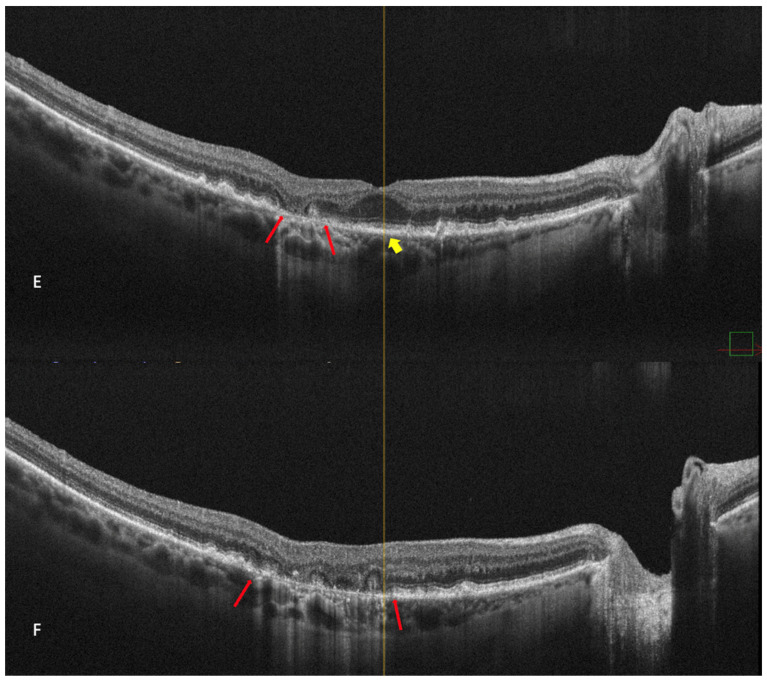
Geographic atrophy. (**A**) cSLO image showing round patches of GA where the choroidal vessels are visible, surrounded by large and medium drusen. (**B**) Green FAF showing confluent hypo-autofluorescent areas. (**C**) DR and (**D**) DL retromode images of GA seen as round patches with homogenous reflectivity where the underlying choroidal vessels are visible. (**E**) SD-OCT showing RPE and outer retinal atrophy temporal to the fovea (red arrows) with foveal sparing (yellow short arrow). (**F**) SD-OCT showing large area of RPE and outer retinal atrophy (red arrows).

In 1970, Gass first used the phrase “geographic zones of atrophy” to refer to “senile macular choroidal degeneration” [39]. Geographic atrophy (GA) is the progressive end-stage form of dry AMD and is represented by a round area where RPE pigment is reduced or absent and where the choroidal vessels are readily apparent (Figure 6A). The smallest area that geographic atrophy can affect is a circle with a diameter of roughly 175 microns [40]. A notable decrease in visual acuity over time in many eyes is linked to geographic atrophy [41]. Even when they were already substantial at baseline, areas of atrophy continue to grow over time [41]. GA has a mean rate of progression of around 1.95 mm^2^/year [42]. Color fundus photography and fundus autofluorescence are two of the main tools used for the diagnosis and monitoring of geographic atrophy [43].

Retromode’s ability to detect and quantify GA was determined by Corradetti et al. by comparison to the classical methods with good and repeatable results [44]. The study compared multiple retinal imaging modalities: retromode deviated-right (DR), retromode deviated-left (DL), confocal color fundus photography (cCPF), and green and blue fundus autofluorescence (G-FAF and B-FAF) using a scanning laser ophthalmoscope [44]. The study revealed the following results using cCFP, G-FAF, B-FAF, retromode DR, and retromode DL, respectively: the mean area of GA was measured as 9.76 mm^2^, 9.75 mm^2^, 9.76 mm^2^, 9.82 mm^2^, and 9.81 mm^2^. Even though there was a numeric difference, and the area measured with retromode DR and DL is larger, the difference is not statistically significant and may be due to a greater sensitivity of the method to detect atrophic changes [44].

The aspect of GA on a retromode illumination image is represented by a pseudo3D round patch with a homogeneous reflectivity and clearly visible hyperreflective choroidal vessels seen under the retina (Figure 6C,D) [44].

#### 3.2.2. Exudative AMD

Wet AMD is represented by neovascularization. It includes several common lesions, such as the presence of intraretinal, subretinal, sub-RPE fluid, RPE detachment, hard exudates, retinal haemorrhages, or disciform fibrotic scars [1].

**Figure 7:**
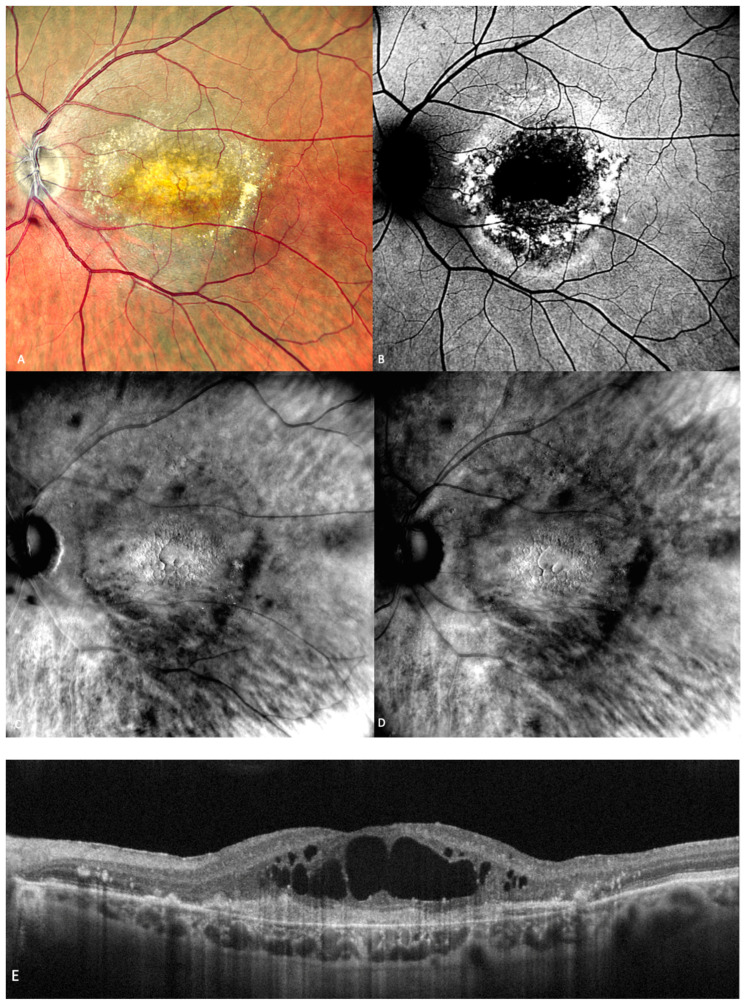
(**A**) The cSLO shows subfoveal fibrosis surrounded by exudates. (**B**) Mottled, irregular autofluorescence pattern with a central hypo-autofluorescent core. (**C**) Retromode DR and (**D**) Retromode DL images showing large, oval, concave, and, respectively, convex cystoid spaces. (**E**) Large intraretinal cystoid spaces seen over an area of subretinal fibrosis on SD-OCT.

Retromode illumination imaging modality’s ability to detect changes in the retina that appear in wet AMD was evaluated by Pilotto et al. [45]. Neuroretinal detachment (NRD) can be seen on the retromode image as an elevated area (62.5% sensibility; 66.7% specificity) [45]. Pigment epithelium detachment (PED) can be detected with retromode illumination as an elevated area (100% sensibility; 69% specificity) [45]. Cystoid macular edema (CME—See Figure 7) is another entity well-detected with retromode illumination (87.5% sensibility; 100% specificity) [45]. Epiretinal membrane was well-detected with retromode (66.7% sensibility; 100% specificity) [45].

Polypoidal choroidal vasculopathy (PCV) is a phenotypic variant of exudative AMD. PCV is usually characterized by subretinal aneurysmal or polypoidal lesions as a result of choroidal neovascularization, frequently accompanied by a branching vascular network. Its diagnosis is important since the lesions present a weak response to anti-vascular endothelial growth factor [46]. Conventionally, the diagnosis is made using indocyanine green angiography (ICGA) and/or SD-OCT.

Zeng et al. investigated the utility of retromode imaging in order to detect and characterize PCV [47]. The results were comparable to ICGA for the polypoidal lesions. Interestingly, retromode was superior to ICGA and fluorescein angiography (FA) for characterizing deep lesions in the RPE and the choroid that appear in PCV. They found no statistically significant differences between retromode and SD-OCT for detecting lesions such as PED, CME, NRD, drusen, or minute granular changes of the RPE. They proposed using retromode as an additional investigation adding up to SD-OCT, ICGA, and FA because it brings comprehensive information about the deep retinal layers and the choroid [47].

CME was described on the retromode images as polygonal or oval cystoid spaces [48] or as a “honeycomb”-like pattern with a central, large, cell-like element (Figure 7) [45].

## 4. Other Macular Diseases Imaged with Retromode

**Figure 8:**
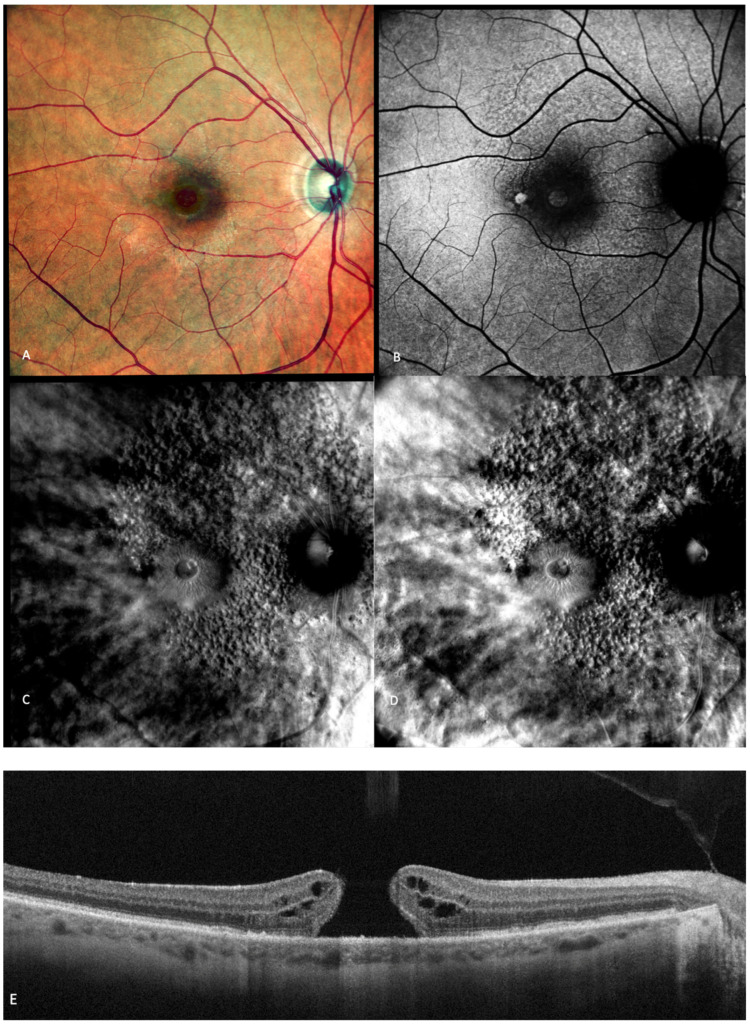
Multimodal imaging of a full-thickness macular hole (FTMH). (**A**) cSLO image showing a red central round area. (**B**) Green FAF showing central hyper-autofluorescence. (**C**,**D**) Retromode DR and DR showing a “single round excavation” [55] and the cystoid spaces around it. (**E**) SD-OCT where the large FTMH is visible.

**Figure 9:**
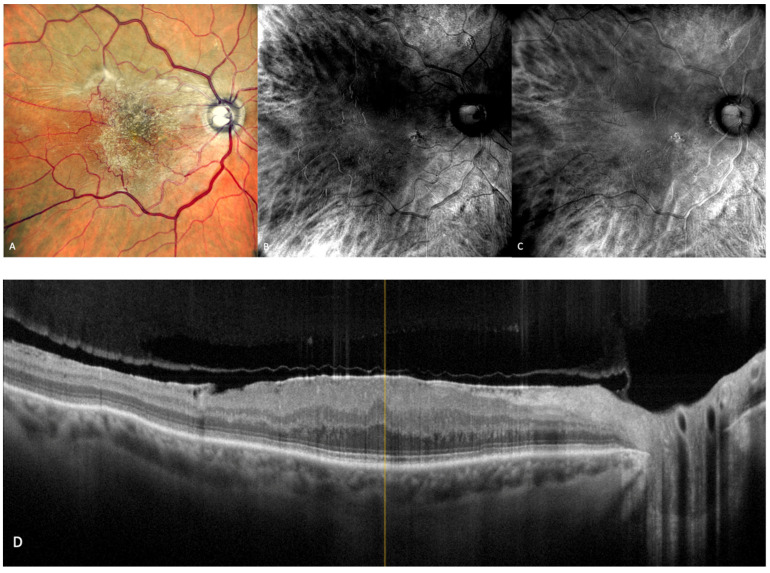
Epiretinal membrane (ERM). (**A**) cSLO image showing ERM folds on the retinal surface. (**B**,**C**) Retromode DR and DL images represent a characteristic “fingerprint” aspect [56]. (**D**) SD-OCT showing the irregular hyper-reflective layer on the retinal surface.

Based only on retromode imaging, it is hard to make a differential diagnosis of different macular diseases. However, retromode has also been proven to be a useful technique in other macular conditions, such as diabetic macular edema [49,50], central serous chorioretinopathy [36,37], cystoid macular edema of diverse aetiology [48], retinal dystrophies [51,52], myopic maculopathy [53,54], full-thickness macular holes [55] (Figure 8), epiretinal membranes [56] (Figure 9), and hydroxychloroquine retinopathy [57]. The scanning laser opthalmoscope retromode imaging was also used to detect subthreshold micropulsed laser spots, which were considered “invisible” before [58].

## 5. Conclusions

Retromode imaging is a relatively new, rapid, non-invasive, and effective retinal-imaging method that is not yet used on a large scale in the medical retina clinic. This imaging method is a comfortable technique, and patients tend to be more compliant with it because it does not necessitate dye injection and because it uses infrared light, which is not as disturbing as other imaging methods using light at the green or blue end of the spectrum.

In the era of multimodality, retromode is an easy-to-use imaging technique that can provide additional information at every stage of the disease. It can help the clinician detect subtle, incipient lesions in the outer retina and the RPE, meaning an early detection of the disease, which is especially important since new treatment options are in the pipeline. Small abnormalities like drupelets can be detected easily with retromode before they are apparent in other imaging modalities. Intermediate AMD lesions can be diagnosed and quantified with this more sensitive technique. Geographic atrophy is also very well-detected and can be monitored and quantified with retromode imaging. Exudative AMD lesions can also be captured with this investigation.

The pseudo-three-dimensional high-contrast images obtained with retromode imaging can provide useful and auxiliary information about the macular region, especially for screening. Retromode certainly can not replace other imaging techniques with well-established utility in the diagnosis and monitoring of age-related macular degeneration, such as SD-OCT or FAF, but it can be a great supplementary tool in the imaging armamentarium of the retinal specialist for everyday use or even in clinical studies.

## Data Availability

Not applicable.
